# Paired Hormone Response Elements Predict Caveolin-1 as a Glucocorticoid Target Gene

**DOI:** 10.1371/journal.pone.0008839

**Published:** 2010-01-21

**Authors:** Marinus F. van Batenburg, Hualing Li, J. Annelies Polman, Servane Lachize, Nicole A. Datson, Harmen J. Bussemaker, Onno C. Meijer

**Affiliations:** 1 Bioinformatics Laboratory, Academic Medical Center, Amsterdam, The Netherlands; 2 Department of Biological Sciences, Columbia University, New York, New York, United States of America; 3 Division of Medical Pharmacology, Leiden/Amsterdam Center for Drug Research and Leiden University Medical Center, Amsterdam, The Netherlands; 4 Department of Biochemistry and Molecular Biology, Life Science College of Nanjing Agriculture University, Nanjing, People's Republic of China; 5 Department of Biochemistry and Molecular Biology, Medical College of Yangzhou University, Yangzhou, People's Republic of China; 6 Center for Computational Biology and Bioinformatics, Columbia University Medical Center, New York, New York, United States of America; INSERM, France

## Abstract

Glucocorticoids act in part via glucocortocoid receptor binding to hormone response elements (HREs), but their direct target genes *in vivo* are still largely unknown. We developed the criterion that genomic occurrence of paired HREs at an inter-HRE distance less than 200 bp predicts hormone responsiveness, based on synergy of multiple HREs, and HRE information from known target genes. This criterion predicts a substantial number of novel responsive genes, when applied to genomic regions 10 kb upstream of genes. Multiple-tissue *in situ* hybridization showed that mRNA expression of 6 out of 10 selected genes was induced in a tissue-specific manner in mice treated with a single dose of corticosterone, with the spleen being the most responsive organ. Caveolin-1 was strongly responsive in several organs, and the HRE pair in its upstream region showed increased occupancy by glucocorticoid receptor in response to corticosterone. Our approach allowed for discovery of novel tissue specific glucocorticoid target genes, which may exemplify responses underlying the permissive actions of glucocorticoids.

## Introduction

Glucocorticoid hormone secretion from the adrenal cortex follows a circadian rhythm and is markedly increased in case of physical or psychological stress, when these steroids can modulate processes in virtually all organs in the body. Glucocorticoids can act permissively to prepare for upcoming stressful challenges and support ongoing stress responses, for instance via increased gluconeogenesis and mental performance. They can also dampen the body's initial reaction to stress, as is the case for anti-inflammatory effects. In addition, glucocorticoids can promote adaptation to stress and the response to subsequent stressors, for example by modulating memory formation. Aberrant glucocorticoid signalling is strongly linked to metabolic, immune, bone, and central nervous system disease, while important pharmacological issues include therapy resistance and side effects [1], [2], [3].

The actions of corticosterone (in rats and mice) and cortisol (in most other mammals) are mediated by glucocorticoid (GR) and – in some tissues – mineralocorticoid receptors (MR). These proteins are members of the nuclear receptor superfamily, and they accordingly act as transcription factors. Regulation of transcription can occur through binding to consensus palindromic sequences in the DNA, known as Hormone Response Elements (HREs), or alternatively through protein-protein interactions in a manner that is independent of direct DNA binding [4]. The latter mechanism may be particularly relevant for the restraining effects of glucocorticoids on activated systems, such as the suppression of NF-kB induced activation of pro-inflammatory genes [5]. However, many important actions involve activation through binding to HREs, as revealed by defects in *dim/dim* mice that are impaired in transactivation at HREs [4], [6], [7].

The understanding of physiological and molecular mechanisms of glucocorticoid action would be advanced by characterization of a larger number of primary HRE-dependent MR and GR target genes than are presently known. Transcriptome analysis using SAGE and microarrays has been performed in various tissues [8], [9], recently in combination with chromatin immunoprecipitation based mapping (ChIP-chip) of genomewide GR occupancy [10]. However, there are limitations to these techniques, such as insufficient abundance of cellular mRNA, tissue heterogeneity, and cell type dependent responsiveness.

The availability of complete genomes has opened the possibility of a complementary approach, namely, the prediction of glucocorticoid responsiveness based on the presence of HREs in the proximity of genes. We have explored this route by studying the occurrence of HREs upstream of 23,391 multi-transcript transcriptional units and 22,658 single mapped transcripts (see Material and Methods). Motivated by empirical evidence that steroid receptors transactivate synergistically when multiple HREs are present in a promoter, we sought to develop a criterion for prediction GR-responsive genes based on a computational analysis of genomewide promoter sequence data and ChIP-chip data for promoter occupancy by GR after glucocorticoid treatment. We concluded that paired occurrence of HREs within 10,000 base pairs upstream of the transcription start site (TSS) at a relative distance less than 200 bp best predicts GR responsiveness. We validated this criterion by performing multiple-tissue *in situ* hybridization on organs harvested 3 hours after corticosterone or vehicle treatment of adrenalectomized mice. We observed that 6 out of 10 genes are indeed up regulated, in a tissue specific manner.

## Materials and Methods

Detailed description of the computational procedures and the source code of all R and Perl scripts is available in supplementary Documents S1 and S2.

### Transcriptional Units and Upstream Regions

To define a set of non-redundant transcriptional start sites (TSS), we used the coordinates of all full-length cDNAs and mRNA sequences (files all_mrna.txt and refSeqAli.txt) as mapped to the March 2005 (mm6) version of the mouse genome available at the UCSC genome annotation project (http://hgdownload.cse.ucsc.edu/goldenPath/mm6). Transcripts whose exons overlapped by one or more base pairs when mapped to the genome sequence were considered to be part of the same transcriptional unit (TU). For purposes of motif analysis (see below), upstream promoter regions were defined relative to TSS. To prevent double counting of motifs, overlapping upstream regions were combined into a single promoter region. All analyses were confined to non-repeat DNA.

### Statistics of Single HRE Consensus Matches

To analyze the spatial distribution of matches to the HRE consensus near transcriptional start sites, we assumed a null model in which matches occur randomly and independently throughout the genome (Bernoulli process), at a density *ρ* equal to their actual genomic density. Thus, the expected number of matches in a single promoter region of length L equals *ρ* (*L-L_HRE_+1*), where *L_HRE_* is the length of the HRE consensus. To quantify whether the observed number of HRE consensus matches for individual promoter regions was significantly larger than expected, P-values were computed using the cumulative Binomial distribution function. These P-values were used as input to the procedure of Benjamini and Hochberg [11] to determine the total number of promoter regions that are significantly enriched by single consensus sites at a false discovery rate (FDR) of 5%.

### Statistics of Paired HRE Matches

Given the single-HRE Bernoulli process defined above, the occurrence of two consecutive HRE consensus matches at distance Δ is again a Bernoulli process, but now at a density *ρ_2_*(Δ) given by
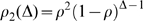
Summing over Δ in a given inter-HRE distance range, and taking into account the lengths of the promoter regions, we computed the expected number of HRE pairs within a distance Δ.

### In Situ Hybridization Experiments

The animal experiment was performed in accordance with the European Communities Council Directive 86/609/EEC and with approval from the animal care committee of the Faculty of Medicine, Leiden University (UDEC number 04052). Sixteen adult male c57bl/6 mice, 25.9±0.4 g (Janvier, France) were adrenalectomized under isoflurane anaesthesia, single housed, and fed with oats containing 400 µg corticosterone or vehicle at t = 0 on the day of the experiment. Mice were decapitated 3 hours after oats administration, trunk blood was collected and organs were dissected and frozen on dry ice. Two mice were killed at 30 minutes after treatment to determine peak levels of hormone. Plasma corticosterone was determined by radio immuno assay (ICN Biomedicals, Costa Mesa, CA). Tissues were cut at 16 µm in a cryostat and collected on poly-L-lysine coated slides. In situ hybridisation with ^33^P end-labelled 45-mer oligodesoxynucleotide probes targeting the first exon of the mRNAs was performed as described [12] probe containing 7 mismatches relative to the anti-sense probes were used as controls. Probe sequences can be found in Table S1 of the additional information. Three exposures (1, 3 and 10 days) of autoradiograms were made to keep the signal in the linear range for all organs, irrespective of absolute level of expression. Autoradiograms were quantified using ImageJ software. Differences between groups were analyzed using Student's t-test. The null hypothesis was rejected at P<0.01.

### Chromatin Immuneprecipitation

A549 human lung carcinoma cell line were grown and maintained in DMEM containing 4.5 g/l glucose, supplemented with 10% Foetal Bovine Serum, penicillin (20 U/mL) and streptomycin (20 ug/mL; all Invitrogen). A day prior treatment, 10×10^6^ cells were seeded on 150 mm dishes. The following day, the cells were treated either with ethanol (vehicle) or 10^−7^M dexamethasone (DEX) for 90 min. and cross-linked to stop reactions. Chromatin immumeprecipitation was performed as described [21]. Fixed chromatin was sheared, yielding fragments of 100–500 bp (20 pulses of 30 seconds; Bioruptor, Diagenode). Immunoprecipitation was performed with either 6µg of GR-specific H300 or normal rabbit IgG (Santa Cruz Biotechnology) overnight at 4°C. After DNA recovery (Nucleospin, Macherey-Nagel), RT-qPCR was performed to study enrichment of the human Cav-1 GRE-1 (GAAACAGAATGTTCT ) (LightCycler FastStart DNA Master PLUS SYBR Green I, Roche), according to manufacturer's instructions. Primers were designed immediately adjacent to the glucocorticoid response element: TGGCTCTTTGGCACTGAGTA (forward), and TGCAGTTTGAAATCCCAACA (reverse). Myoglobulin was used a negative control for GR chromatin occupancy: CCTCACATGGGCAGCTATTT (myoglobin forward); GCTTGTGCAAGTCCAGACAG (myoglobulin reverse). Recovery of DNA was calculated as percentage of input material of the immunoprecipitation.

## Results

### HRE Consensus

Our aim is to predict glucocorticoid-regulated genes based on the spatial distribution of hormone response elements (HREs) in their upstream region. Definition of the HRE consensus sequence is therefore the first step in our analysis. While the canonical HRE is defined as the palindromic sequence AGAACANNNTGTTCT, many variations are possible, particularly in the 3′ half site, both in naturally occurring sites [10], [13], and in parametrically tested variable HREs [14]. Based on these studies, we settled on NGNWCDNNNWGTYCT as a low-stringency definition of the HRE and matched this sequence to both strands to find HREs (coding is according to the IUPAC convention: W = [AT]; S = [CG]; D = [AGT]; Y = [CT]). This sequence is close to the major variants from the TRANSFAC M00205 GRE matrix.

### Single HREs Are Uniformly Distributed Relative to the Transcription Start Site

**Figure 1:**
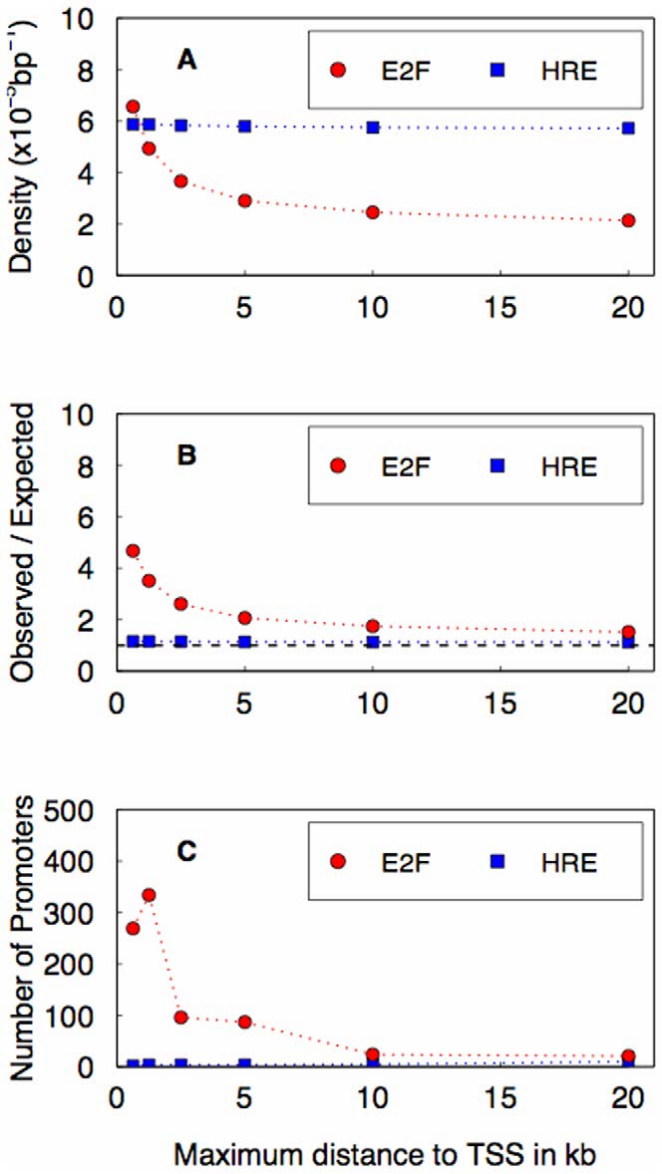
Spatial distribution of matches to the E2F (red) and GR (blue) consensus relative to the transcription start site (TSS). (A) The mean density of response elements over all regions upstream of the TSS for different sizes of the upstream region. The computation of the density was confined to non-repeat DNA. (B) Ratio of observed and expected number of matches versus maximum distance to the TSS. (C) Number of putative target genes at a false discovery rate of 5%, based on enrichment of consensus matches in upstream region, versus maximum distance to the TSS.

Many transcription factors are known to bind preferentially in the vicinity of transcription start sites, including E2F, which binds to TTSGCGC [15]. As a positive control, we determined the distribution of matches to this E2F consensus relative to the TSS (Fig. 1A, red symbols). Indeed, the E2F site occurs more often close to the TSS than at larger distances. This result also indicates that our set of coordinates represents bona fide transcription start sites. Matches to the HRE consensus, by contrast, do not show any enrichment near the TSS (Figure 1A, blue symbols). Concordantly, the observed/expected ratio of consensus matches increases with increasing proximity to the TSS for the E2F, but not for the HRE consensus (Figure 1B). In addition, when enrichment of upstream consensus matches for individual genes is used to define putative cis-regulatory targets at a false discovery rate of 5% (see Methods), a significant number of genes is found only for E2F (Figure 1C). We conclude that the spatial distribution of single HREs cannot be used to define a criterion for selecting genes responsive to GR, consistent with recent data showing that functional steroid receptor binding occurs over large distances up- and downstream of transcription start sites [16].

### Pairs of HRE Matches within 200bp Are Overrepresented Genomewide

**Figure 2:**
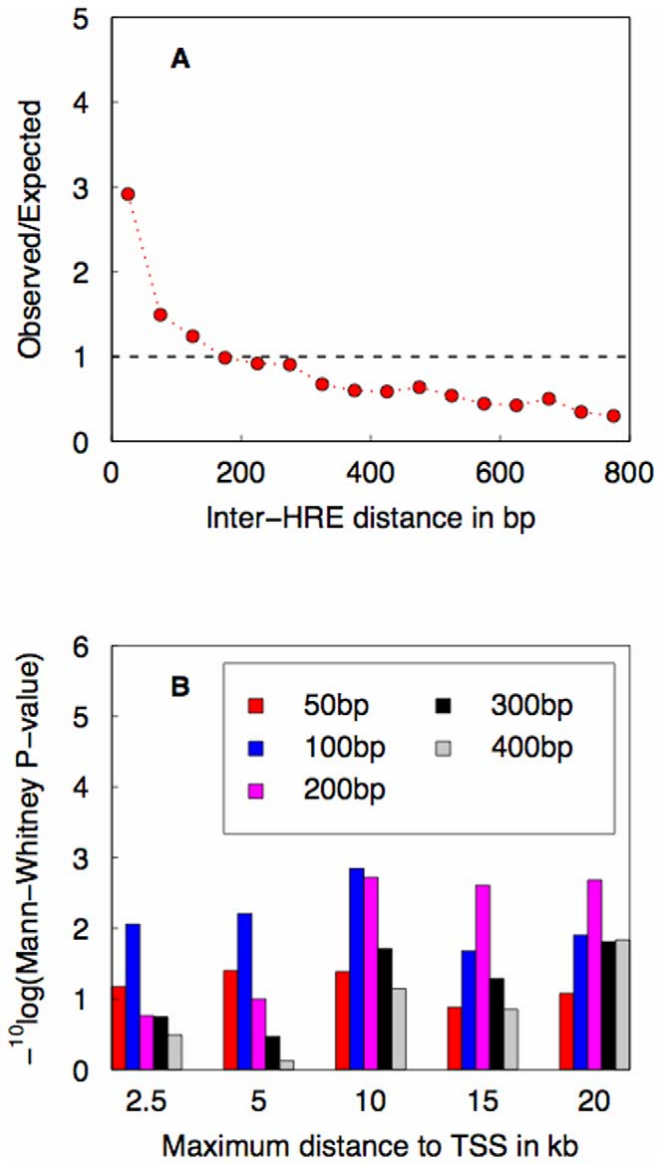
Establishing a criterion for predicting GR target genes based on genomewide analysis of spatial HRE clustering and its association with ChIP-chip data for GR promoter occupancy. (A) Testing for spatial clustering of HREs. Shown is the ratio of observed and expected genomewide density of pairs of HRE matches versus inter-HRE distance. (B) Testing whether paired HREs are predictive of GR binding to upstream regions. Shown is the statistical significance of a non-parametric test for increased occupancy by GR of the upstream regions of putative GR targets, for various combinations of maximum distance upstream of the TSS and maximum inter-HRE distance (indicated by different colours).

The absence of any enrichment of single HREs near the TSS motivated us to investigate the spatial clustering of HREs. It is known that paired occurrence of HREs at relatively close proximity can synergistically stimulate transcription [17], [18], and therefore it may serve as a criterion to predict GR-responsive target genes. Relevant variables in this case are the inter-HRE distance and the distance to the TSS. Assuming a random genomewide spatial distribution of single HREs, the density of pairs of HREs at a given inter-HRE distance can be easily computed (see Materials and Methods). Comparison of the observed and expected genomewide density of HRE pairs shows enrichment at inter-HRE distances shorter than 200 bp (Figure 2A). We conclude from this that there exists evolutionary pressure to maintain such proximal HRE pairs.

### Paired HRE Occurrence Correlates with Measured GR Occupancy

Phuc Le *et al.* [19] used genomewide chromatin-immunoprecipitation (“ChIP-chip”) to measure occupancy by GR in dexamethasone-treated liver samples in the upstream regions of 3291 RefSeq genes important for liver function. We split this set of genes into two parts, putative targets and non-targets, based on whether or not pairs of HRE matches within a given maximum HRE-distance occurred within a given maximum upstream distance from the TSS. We tested whether the observed ChIP enrichment ratios were significantly larger for putative GR targets than for non-targets, using the non-parametric Mann-Whitney test. Consistent with the genomewide sequence analysis result presented in Figure 2A, we found a maximum inter-HRE distance of 200 bp to be optimal for maximum TSS distances of 10kb and higher (Figure 2B).

### A Genome-Wide Scan Predicts 2746 Putative GR Targets

We settled on the criterion that each documented mRNA transcript with an occurrence of two HRE consensus matches within 200 bp from each other and at most 10 kb upstream of its TSS is a putative target of the GR. A genomewide scan identified 2,746 transcripts, corresponding to 553 TUs in the mouse genome. The genomic locations of the HRE pairs corresponding to these putative GR targets are available in GFF and Excel format as Supplementary Information (tables S2 and S3). They can be visualized in the context of the genome annotation by uploading the GFF file to the UCSC genome browser ([http://genome.ucsc.edu).

### Validation

To evaluate the predictive value of paired HREs in genomic regions upstream of transcription start sites, we tested 10 genes from the list of putative GR targets for their responsiveness to glucocorticoids *in vivo*. The genes were selected for heterogeneity of HRE characteristics, such as distance from TSS, sequence variability, and total number of HREs in the cluster. Apart from the number of GREs (which was 2 for the majority of identified clusters) these characteristics showed no particular pattern. We administered corticosterone to adrenalectomized mice in a dose sufficient to occupy both mineralo- and glucocorticoid receptors (peak levels of plasma corticosterone 30 minutes after administration were 28±8 µg/dl). Based on the prediction of primary responses we decapitated animals after 3 hours. Since there can be a considerable degree of cell-specificity in the transcriptional response to glucocorticoids, we harvested multiple organs and evaluated expression and regulation of the mRNAs using multiple tissue *in situ* hybridisation.

**Figure 3:**
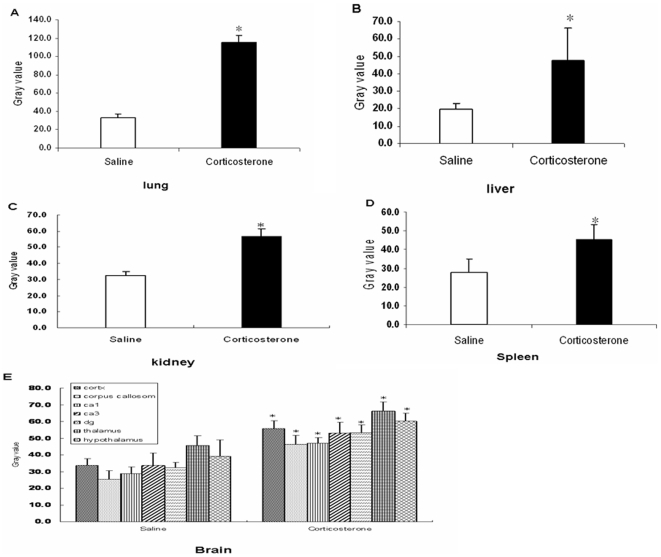
GILZ mRNA expression and its up-regulation three hours after corticosterone treatment of adrenalectomized mice. The average grey value quantified using Image J in the lung (A), liver (B), kidney (C), spleen (D), and brain (E). Bars represent mean ± standard error of the mean of 7 mice/group. Asterisk indicates significant different from control animals.

**Figure 4:**
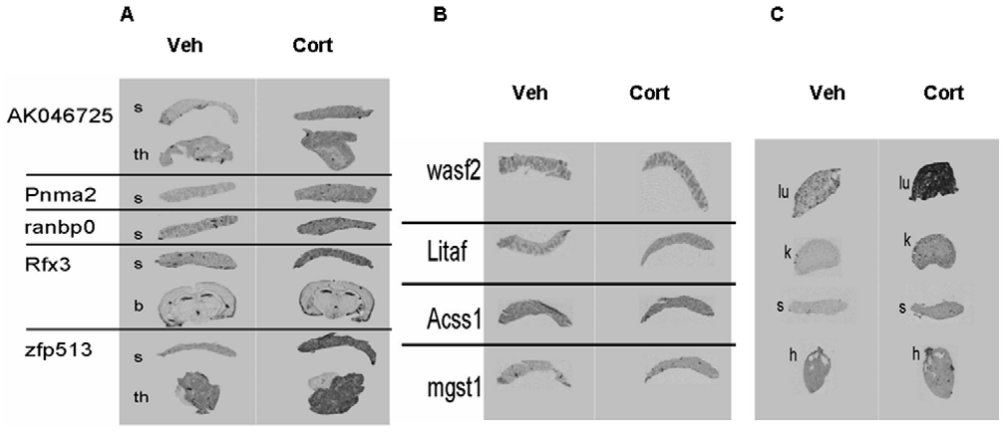
In situ hybridization results for predicted GR targets, 3 hours after corticosterone. Ten genes containing compound HREs in their 10 kb upstream region that were tested. (A) Up-regulation of 5 mRNAs in spleen(s), thymus (th) and brain(b). (B) The hybridization signal in spleen of 4 genes did not show detectable up-regulation in any of the measured organs. (C) *Caveolin-1* mRNA was strongly up regulated in lung (lu), kidney (k), and spleen (s), but not in heart(h).

As a positive control we included measurement of *GILZ* mRNA [20] which was up-regulated in multiple tissues, including lung (Figure 3A), liver (Figure 3B), kidney (Figure 3C), spleen (Figure 3D) and brain (Figure 3E) [21]. All 10 probes against mRNAs containing compound HREs in their flanking region gave a specific signal in at least one tissue; the mismatch control probes gave no signal (not shown). Expression levels of six out of ten mRNA were increased in at least one tissue, 3 hours after corticosterone treatment (Table 1, Table 2, and Figure 4A). Most responsive mRNAs were found in the spleen: 6 out of 10 spleen-expressed mRNAs were up-regulated by 40 to 70%. Four transcripts showed no induction (Figure 4B). The high number of reactive mRNAs in spleen may in part reflect trafficking of lymphocytes upon exposure to corticosterone [2]. However, cresyl violet staining of the sections did not reveal differences is cell density in the spleen.

**Table 1:** Results on multi-tissue *in situ* hybridization experiments.

Organ	Number of genes detected	Number of genes regulated
Spleen	10	6
Thymus	7	2
Lung	2	1
Brain	6	1
Kidney	3	1
Heart	3	0
Liver	5	0
Colon	5	0
Ileum	5	0
Testis	2	0
Muscle	2	0

The number of transcripts expressed before corticosterone treatment, and the number of transcripts differential expressed 3 hours after that treatment, in different mouse tissues.

**Table 2:** Characteristics of the HREs in the validated (upper six) and false positive (bottom 4) predicted GR targets.

Name/Gene symbol/Direct target	GRE-sequence	Distance to TSS(kb)	Inter-GRE distance(bp)
Zinc finger protein 513/*Zfp513*/NM_175311	AGGACTGGTAGTTCTTGCTCATAGTGTCCT	0.6	156
Adult male cortex/530002H17/AK046725	GGCTCGGGAAGTTCTAGGTCATCTTGTCCT	5.2	111
caveolin-1,caveolae protein/*cav*/AK010839/	AGTTCTGAATGTTCTGGAACAGAATGTTCT	2.9	116
0 day neonate thymus cDNA/A430010E21Rik/AK039823 (adjacent to *Rfx3*)	TGCACTCGCTGTCCT (6x)	6.5	5×51
Paraneoplastic antigen MA2/*Pnma2*/NM_175498	AGCTCTGGCTGTCCTGGAACTCAGAGTTCT	2.1	44
RAN binding protein 10/*Ranbp10*/NM_145824/	TGGACTAGTAGTCCTGGGACACTCTGTTCTAGGACAACCAGAGCTAGATCTCTGAGTTCT	4.1	158/105/44
LPS-induced TN factor/*Litaf*/AK150476	GGCACTTCATGTTCTGGCACTTCATGTTCTAGGTCAGGCTGTCCT	6.6	41/120
acetyl-Coenzyme A synthetase 2/*Acss1*/AK004042	AGTTCATGAAGTTCTAGTTCATGAAGTTCTAGTTCATGAAGTTCT	3.5	16/16
WAS protein family member 2/*Wasf2*/AK198823	AGCTCACTCTGTCCTAGCTCTGGCAGTCCT	6.8	114
microsomal glutathione S-transferase 1/*Mgst1*/NM_019946	AGCTCTTTAAGTCCTTGCACTCAGTGTTCT	1.2	61

**Figure 5:**
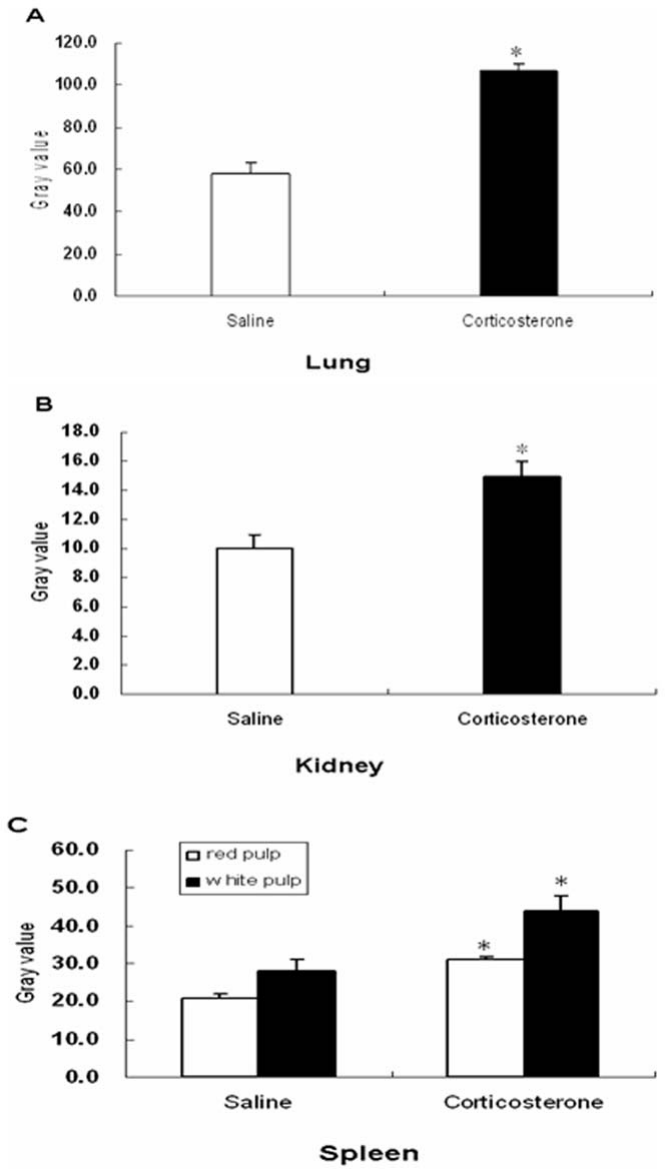
*Caveolin-1* mRNA expression of vehicle versus corticosterone treated animals. The average grey value quantified using image J analysis in lung (A), kidney (B) and spleen(C).Bars represent mean ± standard error of the mean of 7 mice/group. Asterisk indicates a significant different value from that of control animals.

For 3 out of the 10 mRNAs we found significant up-regulation in other tissues that were evaluated, i.e. thymus, lung, kidney and brain (Figure 4A). The highest and most widespread induction was observed for caveolin-1 mRNA, which in lung, kidney, and spleen was up-regulated by 15–50%, while not in heart. (Figure 4C and Figure 5).

**Figure 6:**
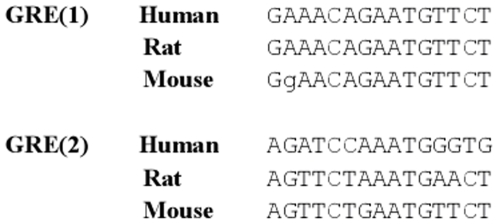
Alignment of *caveolin-1* putative GREs in human, rat and mouse genome. Mouse *caveolin-1*GRE-(1) is located 1526 bp before TSS; only one nucleotide is different in the rat and human genome. GRE(2) is located at position −1643. The left half-site of second GRE in mouse is conserved in the rat, but the sequence is not well conserved in human.

**Figure 7:**
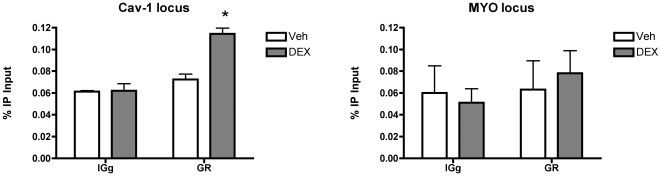
Dexamethasone leads to recruitment of GR near the cave*olin-1* GRE-1. A significant 2-fold enrichment of GR was observed for caveolin-1, but not the negative control region in the myoglobin gene, after treatment of A549 cells with dexamethasone (10^−7^ M). n = 4 dishes per condition.

As an additional validation we performed chromatin immune precipitation on the GRE upstream of the caveolin-1 gene in a human lung carcinoma cell line (Figures 6, 7). Dexamethasone treatment led to a modest (∼2-fold) but significant enrichment of the GRE-associated DNA after precipitation with the GR antibody, but not with IgG. For the negative control DNA, the myoglobin locus, no enrichment was observed.

## Discussion

Through computational analysis of the spatial distribution of the GR consensus binding motif in upstream promoter regions, and its relationship with GR occupancy as measured using ChIP-chip, we have developed a simple criterion that allows for prediction of *in vivo* glucocorticoid responsiveness based on the 10kb sequence upstream of the transcriptional start site of each mRNA transcript annotated in the mouse genome. Testing 10 genes chosen from the list of putative targets, we identified six novel corticosterone-responsive genes. Most regulated mRNAs were found in the spleen. C*aveolin-1* and two other genes showed regulation in other tissues.

A requirement for paired occurrences of the HRE within ∼200bp was part of our final criterion for predicting target genes. We found that larger or smaller inter-HRE distance cut-offs gave worse correspondence with genome-wide ChIP-chip measurements of GR occupancy. The length scale of ∼200bp is similar to that of nucleosomal DNA, which is ∼147bp. It is tempting to speculate that the simultaneous binding of two GR molecules to distinct but proximal HREs helps displace nucleosomes that block access to the DNA. Such nucleosome-mediated cooperativity has been observed in other contexts [22], [23], and it is known that cis-regulatory elements occurring within clusters of transcription factor binding sites are more likely to be functional *in vivo* [24]. For 73 genes, we found more than one HRE pair in their upstream region.

The strongest responsive gene we identified is *caveolin-1*. The gene was up regulated in lung, kidney, and spleen; in most other tissues expression was too low to be quantified in a reliable way. The HRE matches that led to identification of *caveolin-1* as a GR target occur at position −1526 bp upstream of the cav-1 transcription start site. Consistent with the notion that functional GR-binding sequences are evolutionary conserved [16], we found that the GREs upstream of *caveolin-1* in mouse are conserved in rat (one mismatch in each HRE) and, to a lesser degree, in human (Figure 6). The surrounding sequences of the two GREs were also conserved. ChIP data on A549 cells lend support to the functionality of this GRE in multiple species. Unfortunately, the ChIP methodology may not have sufficient resolution to distinguish between the two GREs, given the fragment length of up to 500 bp. Earlier studies have implicated the androgen receptor (AR) in human prostate cancer cells [25] and the progesterone receptor (PR) in breast cancer cells [26] as stimulators of *caveolin-1* expression. The AR and PR can both recognize the low-stringency HRE sequence we used when tested in isolation [14], and the conserved HREs may mediate the effects of PR and AR in vivo.

*Caveolin-1* has recently received substantial attention [27]. Caveolines (cav-1, cav-2, cav-3) are major constituents of caveolae, which are small invaginations of the plasma membrane that function as vesicular transporters [28]. Among them *caveolin-1* was the first to be discovered and has been characterized most extensively [29], [30]. *Caveolin-1* forms a major component of caveolae, which are involved in sub-cellular transport, cholesterol homeostasis, as well as signal transduction. *Caveolin-1* may play a role in non-genomic steroid receptor mediated effects [31], [32] and has been implicated in several types of cancer, metabolic, and cardiovascular disease, and via its link with cholesterol transport in Alzheimer's disease [33]. Interestingly, *caveolin-1* knockout mice suggest a permissive function of the protein: while its absence leads to disappearance of caveolae in many organs, the mice are viable and fertile. However, these animals are much more susceptible to numerous challenges, which may lead to development of diabetes and cancer. *Caveolin-1* seems to be a good example of a gene that is regulated by corticosterone in absence of challenge and involved in permissive hormone effects.

With respect to the other hits from our genomic search, the simple criterion we applied necessarily must have yielded numerous false negative results. A number of known regulated genes (*e.g. PNMT*, *GILZ*) were simply lacking either because functional HREs occur singly (or as pairs at an inter-HRE distance >200 bp), do not match our consensus, are located in intronic regions [16], [34], [35], [36], are further upstream than 10 kb [16], or occur in repeat DNA. Also, only the most upstream transcription start sites of genes were considered. The frequency of occurrence of the low-stringency HRE that we used is on the order of 10^−5^ bp^−1^, and selection of promoters with single elements will likely result in a high false-positive rate. Yet, the validity of our HRE definition is supported by the fact that the nucleotide sequence of the HREs occurrences for our responsive genes covers almost the entire range of our low-stringency consensus. The predictive value of the compound (paired) HREs may increase by the use of weight matrices [37], [38] selection for cross-species conservation (as present in the *cav-1* gene), and taking into account the presence of non-HRE binding sites of co-factors [19], [39].

Some of the changes in transcript levels that we observed may be secondary effects, i.e. changes in gene expression that are induced by the product of a primary corticosterone-responsive gene. However, we interpret our set of predicted GR targets as strongly enriched for primary responsive genes, given the selection criterion, the relatively short time after treatment, and the fact that the mRNAs were always up regulated, as would be expected from the compound HRE mediated action. Lastly, the HREs we identified do not necessarily mediate the corticosterone-induced transactivation. Some strongly steroid responsive genes such as *PNMT* and *GILZ* contain multiple HRE-like sequences, not all of which are necessary for induction by hormones [10], [13]. Our selection criterion may simply select for such HRE-rich genomic stretches. Additional evidence for involvement of particular HREs could come from studies in cell lines using chromatin immunoprecipitation of GR/MR, and mutation of candidate HREs in reporter studies.

Effects of glucocorticoids depend heavily on the physiological state of the animal. For example, mRNA induction in liver, a classical target tissue, is very weak in mice that are well fed, compared to mice that are starved [19]. This may explain why we did not observe any effect of corticosterone on the putative target genes in the liver. Our results indicate that the spleen is highly responsive to acutely elevated corticosteroid levels. While we did not determine the exact cell type in which regulation occurred, it is tempting to link these findings to the immune system, which has long been recognized as a target of corticosteroids in delayed anti-inflammatory effects [40]. However, acute exposure to glucocorticoids can have immune-enhancing effects, which have been interpreted as permissive [2], [41]. Corticosterone treatment has been shown to allow higher production of anti-CD3 induced IL-4 production in spleen lymfocytes [42] and can acutely enhance splenic T cell proliferation [43]. The products of up-regulated mRNAs as identified under the present conditions may mediate such permissive effects. On the other hand, immune-suppressive effects like apoptosis can also depend on induction of target genes via HREs [4].

Our decision to look for compound HREs was based on the expectation of a large response due to transcriptional synergy. The set of currently identified targets may represent examples for some promoter (paired HRE) dependent phenomena that have been described in experimental settings, but do not have clear physiological interpretation, such as SUMO-ylation-dependent synergistic activation [44] differential coregulator efficiency [45], [46], and receptors with mutated dimerization domains that retain transactivation potential at compound HREs [4], [13], [47].

In conclusion, we have demonstrated successful prediction of glucocorticoid-regulated genes *in vivo* from a genome wide screen for compound HREs in regions upstream of transcription units. Our findings suggest prominent and acute effects of corticosterone on the spleen, and reveal caveolin-1 to be strongly regulated by corticosterone in several tissues. While future searches will need to incorporate additional criteria, including sequence conservation, our results suggest a new approach to understand stress regulated disease in the context of the whole animal, rather than single cell lines [48].

## Supporting Information

Table S1 Probe sequences used (Word doc), Probes used for ISH validation
(0.05 MB DOC)

Table S2 All genomic HRE pairs with mm6 annotation (Excel file)
(2.99 MB XLS)

Table S3 All genomic HRE pairs in GFF format. The HREs can be visualized in the context of the genome annotation by uploading the file to the USCS genome browser available at http://genome.ucsc.edu/.
(0.60 MB TXT)

Document S1 Detailed description of computational procedures (word doc)
(0.05 MB DOC)

Document S2 Source code of all R and Perl scripts used in the analysis
(4.54 MB GZ)
