# Supplementary material for: Paired Hormone Response Elements Predict Caveolin-1 as a Glucocorticoid Target Gene
Source: PLoS One. 2010 Jan 21;5(1):e8839. doi: 10.1371/journal.pone.0008839 (PMC2809115; doi:10.1371/journal.pone.0008839)
Supplement: Table S1 — Probe sequences used (Word doc), Probes used for ISH validation (0.05 MB DOC) [file pone.0008839.s001.doc]

**Probes used for validation**

**Zfp513**

Match: 5' CTGCACTTCCCGTACTCGATGACCCTCAGGTTCCTTTAATACTCT

mismatch: CTGCcCTTCCaGTACTaGATGAaCCTCAtGTTCCgTTAATcCTCT

**Litaf1**

Match: 5' TTTGCAGTTGGGGCAGTAGTGGTCCACATCCTGTAGGGCGTCTAC

Mismatch: TTgGCAGTgGGGGCcGTAGTtGTCCAaATCCTtTAGGGaGTCTAa

**Pnma2**

Match: 5' TCCATAAGTTCCACTAAGACAACACTGGTGTTGTCCTGCTTCTGG

Mismatch: TCCAgAAGTTaCACTAcGACAAaACTGGgGTTGTaCTGCTgCTGG

**Acss1/Acas21**

Match: 5' TCCTCATCACTTTCCCAGATCTGGTTTTGGGGAGACGCTTCACCA

Mismatch: TCCTaATCACgTTCCCcGATCTtGTTTTtGGGAGcCGCTTaACCA

**Cav1**

Match: 5' TTCAAAGAGTGGATCGCAGAAGGTATGGACGTAGATGGAGTAGAC

Mismatch: TTaAAAGAtTGGATaGCAGAcGGTATtGACGTcGATGGcGTAGAa

**Wasf2**

Match: 5' ACAGGATGGTGGCCACATCATTGCCCACCACATCACGCTTCTCTT

Mismatch: AaAGGATtGTGGCaACATCcTTGCCaACCACcTCACGaTTCTCgT

**Mgst1**

Match: 5' TTGTTGGTTATCCTCTGGAATGCGGTCGCAGAGCTCATGAACATC

Mismatch: gTGTTGtTTATCaTCTGGcATGCGtTCGCAtAGCTCcTGAACcTC

**Ranbp10**

Match: 5' CATCGGCCTCTCCCAGGGAAACCTCCGTGTCCATTACAAAGGTAT

Mismatch CcTCGGCaTCTCCaAGGGAcACCTCaGTGTCaATTACcAAGGTcT

**AK046725**

Match:5' catggttgctagtagacaaatcccaactgcggagccattcaagct

Mismatch: catTgttgcGagtagCcaaatAccaacGgcggaTccattAaagct

**Rfx3**/**AK039823**

Match: 5' TTTTGGCCCGAGGCTCTGAAGAATCATCCAGATCTTCATCCGTCT

Mismatch: gTTTGGaCCGAGtCTCTGcAGAATaATCCAtATCTTaATCCGgCT
