# Supplementary material for: Paired Hormone Response Elements Predict Caveolin-1 as a Glucocorticoid Target Gene
Source: PLoS One. 2010 Jan 21;5(1):e8839. doi: 10.1371/journal.pone.0008839 (PMC2809115; doi:10.1371/journal.pone.0008839)
Supplement: Document S1 — Detailed description of computational procedures (word doc) (0.05 MB DOC) [file pone.0008839.s004.doc]

**Supporting information (S4)**

**Aim**

This report describes the data and software tools that are required for reproducing the results presented in the main text. The results include the plots in Figure 1 and 2, the Transcriptional Units (TUs) and the predicted Hormone Response Elements (HREs) and Glucocorticoid Responsive (GR) genes (Table S2 (S3)). The materials including results) can be downloaded from the supporting info section (zipped tar file S4.tar.gz).

**Requirements**

**Software and hardware**

The tools work under operating system Linux, UNIX and MacOS X in which a recent version of statistical computing environment R (e.g. 2.8) and programming language Perl are installed. Install the bioconductor package (library) *multtest* to include the FDR procedure by Hochberg and Benjamini as part of R.

The tools, data and plots require 6 Gbyte of diskspace.

**Data**

The following public available data must be downloaded:

- Files with chromosome DNA (chr*.fa) (genome.ucsc.edu, mm6)
- Gene annotation files refSeqAli.txt and all_mrna.txt (genome.ucsc.edu, mm6, database)
- The file 10.1371_journal.pgen.0010016.sd002.csv, which is the supporting dataset S2 of article Phuc Le et al. 2005.

**Tasks required for reproducing the figures and supplementary data sets**

First, place the public available data on the appropriate directories:

- The files chr*.fa, fa and 10.1371_journal.pgen.0010016.sd002.csv are placed in directory mm6
- Gene annotation files refSeqAli.txt and all_mrna.txt are put in directory TUs

Secondly, run script pipelineTUs.sh on directory TUs to construct TUs.

Finally, run shell scripts 50kAll.sh and 50kNonRpeats in directory 50000, and TwoSampleTestGR.sh in directory gr.

**Implementation of a pipeline**

For each figure or table, a directory is made. In that directory a pipeline for producing the figure or table is implemented as a master Bourne shell script in which several Perl and R scripts process the data. The shell variables DataDir, RDir, PerlDir and ChromDir point to the subdirectories that host TUs, chromosome DNA sequences, R and Perl scripts respectively. We plotted the graphs with R.

In the following sections, the pipelines (shell and R scripts) and their components are described.

**FIGURE 1**

Data for plot 1A and 1B are produced by script Amkfdr.sh. This script makes use of the in-house Perl script DownRefSingleFDRestimation.pl. It produces the following output files:

- A file called AllNonOverlappingMerged_[upstream] where the number in the brackets refers to the size of the upstream region. It contains the number of consensus matches and non-repeat nucleotides for all upstream regions i.e. the data plotted in Figure 1A.
- AllLengthMatchesHRE.txt_{nonrepeats}_[upstream]

This file contains info about consensus matches and non-repeats per merged upstream region. These data are used to plot figure 1C.

- AllCandidateFirstExonsTUs.txt_[upstream]

This file contains extra info about first exons within TUs.

Input data are stored in the files:

- [chrom]_mm6_Merged_Exons_${strand}_sorted.gff_filtered.tus

TUs and their transcripts per chromosome (see construction of TUs)

- UniqueGenes: genes that do not have a paralog on the same combination of chromosome and strand.
- mm6_Exons_bothstrands_all_sorted.gff_filtered (see construction of TUs)

This file contains info about locations of first exons per TU.

- The files chr?.fa contain chromosome DNA sequences (mm6, genome.uscs.edu).

The next step produces the plots of Figure 1 by applying the following shell scripts:

- Shell script Fig1A.sh under sub directory Fig1_data, produces the data for Figure 1A. The data are stored in file barscols.dat.
- Script Fig1B.sh under subdirectory Fig1_data, produces the data for Figure 1B (Fig1Bbarconstcols.dat).
- Shell script mkANE2F.sh (mkAHRE.sh) selects E2F (HRE) enriched promoters using an FDR-based method. Perl script Fig1C.pl under subdirectory Fig1_data, produces the data for the plots (Fig1CcorrNcols.dat).

The plots are made with R script Fig1article.R under directory Fig1.

**FIGURE 2**

**Pipeline for Figure 2A**

Bourne shell script pipelineGenomeFDR.sh is located in directory genome. It is the implementation of the pipeline that produces the genome-wide distribution of observed and expected HRE pairs at different inter-HRE distances.

First step of this pipeline is the computing of the genome-wide density of HREs in non-repeat DNA. For this purpose we have a script called CountBpReps.pl that computes the total number of HREs and non-repeat bases (numbers are stored in file BpValues).

We applied NFmmpairsCaseSens.pl to match chromosome DNA sequence to our consensus sequence for HRE. HRE pair characteristics (inter-HRE distance, location of HREs, their orientations) are listed as output for each chromosome in file HREPairsOnNonRepeats.txt.

The script FDRcaseSense.pl processes the information on HRE pairs listed in file HREPairsOnNonRepeats.txt, to obtain the number of observed and expected HRE pairs. We used the total number of non-repeat nucleotides (BpValues) to compute the genome-wide density of HRE pairs in non-repeat DNA.

**Pipeline for Figure 2B: GR occupancy analysis**

The shell scripts TwoSampleTestsGr.sh in directory GR implements the pipeline for producing data of Figure 2B.

Its input (see Requirements for details):

- File 10.1371_journal.pgen.0010016.sd002.csv under directory mm6

(GR binding data and gene annotation)

- Files refSeqAli.fa_header.gz and all_mrna_Gene.fa.gz.

(Gene annotation of transcripts obtained from gene bank)_

- The file at subdirectory mm6 50000mm6_both_sequence_30kb0kb.txt_psingle_2clusterID_testnonrepeats (Output of 50kNonRepeats.sh under directory 50000).

The pipeline consists of the following steps:

- It first calls the Perl script GenesToRatiosB.pl. Its input is refSeq headers and 10.1371_journal.pgen.0010016.sd002.csv. The output file is PromoterStuff.inp that contains probe id, the observed binding ratio and targeted refSeq gene ID.
- On the basis of given maximum distance upstream of TSS and given maximal inter-HRE distance, GR genes have been predicted. We subsequently employed the Perl script AnnotateGR.pl to make a GFF file and Perl scripts getdirectnms and unique.pl to select unique refSeq genes (stored in file overlap_L[size upstream region]_D[inter-HRE distance]).
- We subsequently sample a group of binding ratios belonging to the predicted refSeq GR genes and a group of remaining ratios. We applied a one-sided two-sample Wilcox test (R function *wilcox.test()*) to these samples to obtain a test p-value (see main text).
- We repeated the previous two steps but for different combinations of inter-HRE distance and maximal distances upstream TSS. The results have been plotted in figure 2B.

The output of both pipelines is plotted in figure 2 (see subdirectory Fig2) using R-script NFig2article.R

**Construction of TUs**

The Bourne shell script pipelineTUs.sh in directory TUs automates the construction of TUs and TSS. Its input data includes the files all_mrna.txt and refseqali.txt (mm6 genome.ucsc.edu).

The following scripts accomplish this task:

- Reftogff.pl: converting all_mrna.txt and refseqali.txt into GFF formatted files
- ExonCounting.pl: numbering exons starting from first exon till terminal exon.
- unix command grep in combination with splitinchroms.pl to group exons according to chromosome strands.
- Script mergePredProgGenes.pl to merge overlapping exons on each chromosome strand.
- Script MakeClasses.pl to cluster overlapping transcripts into TUs using a equivalent class algorithm.

Finally, the shell script pipelineTUs.sh copies the output files UniqueGenes, chr[chrom]_mm6_Merged_Exons_[strand]_sorted.gff_filtered.tus, and mm6_Exons_bothstrands_all_sorted.gff_filtered to mm6.

**Predicted HRE pairs and their downstream GR genes**

Subdirectory S2_S3 contains the script S2S3.sh that produces the genomic HRE pairs with mm6 annotation in GFF format (Table S3, HREs_S3.gff) and in tabulated ASCII format (HREs_S2.txt). The ASCII file is exported to excel file by program excel (Table S2). This script uses as input the file: 50000mm6_both_sequence_30kb0kb.txt_psingle_2clusterID_testall, which is produced under directory 50000 by shell script 50kbAll.sh and placed in directory mm6. It calls the perl script AllFullMakePredictionsGFF.pl to annotate all pairs of HREs within 50kb upstream of each first exon in terms of their chromosome location, DNA contents and targeted transcripts (50000mm6_both_sequence_30kb0kb.txt_psingle_2clusterID_testall). This perl script requires as input the following files (output of pipelineTUs.sh, mm6):

- chr[chrom]_mm6_Merged_Exons_[strand]_sorted.gff_filtered.tus (in mm6): contains the transcriptional units with transcripts whose exons overlap.
- mm6_Exons_bothstrands_all_sorted.gff_filtered (in mm6): contains the chromosome locations of the exons (including the first exon) of all transcripts.
- chr[chrom].fa (genome.ucsc.edu, mm6): DNA of the pertinent chromosome.
